# TRIM21 accelerates ferroptosis in intervertebral disc degeneration by promoting SLC7A11 ubiquitination and degradation

**DOI:** 10.1515/biol-2025-1216

**Published:** 2025-12-30

**Authors:** Wei Yu, Yong Liu, Feng Zhou

**Affiliations:** Department of Orthopedics, Jiangyin People’s Hospital, Jiangyin, Jiangsu, China

**Keywords:** intervertebral disc degeneration, ferroptosis, TRIM21, SLC7A11, ubiquitination

## Abstract

Intervertebral disc degeneration (IVDD) is a common cause of chronic low back pain, and ferroptosis – an iron-dependent form of cell death – has been linked to oxidative stress-induced damage in nucleus pulposus cells (NPCs). This study focuses on the role of TRIM21, an E3 ubiquitin ligase, in regulating ferroptosis during IVDD. TRIM21 expression was analyzed in clinical IVDD samples and tert-butyl hydroperoxide (TBHP)-treated NPCs. Ferroptosis was measured by assessing cell viability, Fe^2+^/ROS accumulation, and lipid peroxidation. The mechanism was investigated through Co-immunoprecipitation (Co-IP), ubiquitination assays, and cycloheximide (CHX) chase experiments. Results revealed that TRIM21 was significantly upregulated in degenerated discs. Silencing TRIM21 alleviated TBHP-induced ferroptosis, reducing iron overload, ROS, and lipid peroxidation, while restoring antioxidant activity and modulating ferroptosis-related proteins. Ferrostatin-1 rescued TRIM21-overexpression-induced ferroptotic injury. Mechanistically, TRIM21 bound SLC7A11 and promoted its K48-linked ubiquitination and proteasomal degradation. SLC7A11 knockdown reversed the protective effect of TRIM21 silencing. Thus, TRIM21 exacerbates IVDD by driving ferroptosis through ubiquitin-mediated degradation of SLC7A11, highlighting its potential as a therapeutic target.

## 1 Introduction

Intervertebral disc degeneration (IVDD) is a prevalent musculoskeletal disorder and a leading cause of chronic low back pain, affecting millions of individuals worldwide [1]. Epidemiological studies indicate that IVDD-related symptoms occur in approximately 40 % of adults over 40 years of age, with the prevalence increasing markedly with age [2]. The pathogenesis of IVDD involves the progressive structural and functional deterioration of the intervertebral disc (IVD), characterized notably by the loss of nucleus pulposus cells (NPCs) – a cell population essential for maintaining disc homeostasis [3]. Oxidative stress, driven by excessive production of reactive oxygen species (ROS), is a key contributor to NPC dysfunction and death, thereby accelerating IVDD progression [4]. Among various forms of regulated cell death, ferroptosis – an iron-dependent process driven by lipid peroxidation – has recently been identified as a critical mechanism in IVDD [5], 6]. Nevertheless, the specific molecular regulators that connect oxidative stress to ferroptosis in NPCs remain poorly defined.

Ubiquitination is a highly dynamic post-translational modification orchestrated by a sequential enzymatic cascade involving E1 (activating), E2 (conjugating), and E3 (ligase) enzymes [7]. E3 ubiquitin ligases serve as the central regulators of this system, dictating substrate specificity and the topology of ubiquitin chains, thereby enabling precise control over protein stability, activity, localization, and interactions [8]. Tripartite motif-containing protein 21 (TRIM21), a member of the TRIM family, is an E3 ubiquitin ligase recognized for its diverse functions in immune regulation, protein degradation, and cellular stress responses [9]. It mediates the ubiquitination of target proteins, typically leading to their proteasomal degradation, and consequently influences their stability, localization, and function [10]. While TRIM21’s role in autoimmune diseases and viral infections is well-established [11], 12], emerging evidence indicates its involvement in a broader spectrum of pathological conditions, including cancer, neurodegeneration, and metabolic disorders [13]. Notably, TRIM21 has also been implicated in oxidative stress responses through the modulation of key signaling pathways [14]; however, its specific function in IVDD and the regulation of ferroptosis remains entirely unexplored.

Solute carrier family 7 member 11 (SLC7A11), a key component of the cystine/glutamate antiporter (system Xc^−^), plays a central role in suppressing ferroptosis. It mediates cystine uptake for glutathione (GSH) synthesis, thereby maintaining cellular redox balance [15], 16]. Downregulation of SLC7A11 impairs cystine import, resulting in GSH depletion, accumulation of lipid peroxides, and ultimately, ferroptotic cell death [17]. Since TRIM21 regulates protein stability via ubiquitination, we hypothesized that TRIM21 might promote IVDD progression by driving the ubiquitin-mediated degradation of SLC7A11 and facilitating ferroptosis in NPCs under oxidative stress.

In this study, we aimed to investigate the role of TRIM21 in IVDD pathogenesis, with a specific focus on its regulation of SLC7A11-mediated ferroptosis in NPCs. By elucidating the molecular mechanism through which TRIM21 contributes to oxidative stress-induced disc degeneration, our findings may offer new insights into potential therapeutic strategies for IVDD.

## 2 Methods

### 2.1 Clinical study

A total of 26 patients with confirmed IVDD and 26 age- and sex-matched healthy controls were enrolled in this study. The diagnosis of IVDD was established based on clinical symptoms and imaging findings (MRI or CT). Exclusion criteria encompassed systemic inflammatory diseases, malignancies, recent infections, or the use of immunosuppressive medications. Peripheral blood samples (5 mL per subject) were collected and centrifuged. The resulting serum was aliquoted into sterile cryovials and stored at −80 °C until subsequent analysis.

**Informed consent:** Informed consent has been obtained from all individuals included in this study.

**Ethical approval:** The research related to human use has been complied with all the relevant national regulations, institutional policies and in accordance with the tenets of the Helsinki Declaration, and has been approved by the Ethics Committee of Jiangyin People’s Hospital.

### 2.2 Cell culture and treatment

Human nucleus pulposus cells (NPCs) were obtained from ScienCell Research Laboratories (Cat. No. 4800, Carlsbad, CA, USA) and maintained in NPC Medium (ScienCell) supplemented with 2 % FBS, 1 % NPC Growth Supplement, and 1 % penicillin/streptomycin at 37 °C in a 5 % CO_2_ atmosphere. To establish an oxidative stress model, NPCs were exposed to 100 μM tert-butyl hydroperoxide (TBHP; Sigma-Aldrich, St. Louis, MO, USA) for 4 h.

In inhibitor pretreatment experiments, NPCs were treated with 500 μM of the apoptosis inhibitor TUDCA (Tauroursodeoxycholic acid), 20 μM of the necroptosis inhibitor Necrostatin-1 (NSA), or 10 μM of the ferroptosis inhibitor Ferrostatin-1 (Fer-1) for 12 h prior to TBHP induction. All inhibitors were sourced from MedChemExpress (MCE, Monmouth Junction, NJ, USA).

### 2.3 Cell transfection

Short hairpin RNA (shRNA) constructs targeting TRIM21 (shTRIM21) and SLC7A11, along with a non-targeting shRNA negative control (shNC), were supplied by Shanghai GenePharma Company. These plasmids were transfected into NPCs using Lipofectamine 3000 reagent (Invitrogen, Carlsbad, CA, USA) according to the manufacturer’s instructions.

### 2.4 Cell viability

Cell viability was assessed using a Cell Counting Kit-8 (CCK-8) assay (Beyotime, Shanghai, China). Briefly, NPCs were incubated with 10 μL of CCK-8 reagent per well for 2 h at 37 °C. The absorbance at 450 nm was then measured using a microplate reader (Thermo Fisher Scientific, Waltham, MA, USA).

### 2.5 Quantitative real-time PCR (qPCR)

Total RNA was extracted from cells using a commercial RNA isolation kit. cDNA was then synthesized from the extracted RNA using the RevertAid First Strand cDNA Synthesis Kit. qPCR was performed on an ABI 7500 Real-Time PCR System (Thermo Fisher Scientific). The relative gene expression levels were calculated using the 2^−ΔΔCT^ method, with GAPDH serving as the internal reference gene for normalization.

### 2.6 Measurement of Fe^2+^, reactive oxygen species (ROS), malondialdehyde (MDA), superoxide dismutase (SOD), and glutathione (GSH) levels

Intracellular ROS levels were measured using a ROS Fluorometric Assay Kit (EEA019, Thermo Fisher Scientific). The concentrations of Fe^2+^, MDA, SOD, and GSH in NPCs were determined with corresponding commercial detection kits (Solarbio, Beijing, China). All assays were performed in strict accordance with the manufacturers’ protocols.

### 2.7 Immunoblotting

Total protein was extracted from cells using standard methods, and the protein concentration was quantified. Subsequently, 50 μg of protein per sample was separated by 10 % SDS-PAGE and electrophoretically transferred onto a PVDF membrane. After blocking with 5 % non-fat milk, the membrane was incubated with specific primary antibodies (1:1,000 dilution) overnight at 4 °C, followed by incubation with a HRP-conjugated secondary antibody (Abcam, Cambridge, MA, USA) for 2 h at room temperature. Protein bands were visualized using a ChemiDoc™ XRSC imaging system (Bio-Rad, Hercules, CA, USA). The primary antibodies used in this study were as follows: anti-ACSL4 (ab155282, Abcam), anti-GPX4 (ab125066, Abcam), anti-TFRC (ab214039, Abcam), anti-TRIM21 (ab207728, Abcam), anti-SLC7A11 (ab307601, Abcam), anti-Ubiquitin (ab134953, Abcam), anti-HA (ab18181, Abcam), anti-His (ab18184, Abcam), anti-Flag (ab125243, Abcam), and anti-β-actin (ab8226, Abcam).

### 2.8 Immunoprecipitation (IP) and co-IP

NPCs were collected and lysed in ice-cold IP buffer supplemented with protease and phosphatase inhibitors under gentle agitation. The lysates were centrifuged to remove cellular debris, and the resulting supernatants were incubated overnight with 5 µg of the respective target antibody at 4 °C. Protein A/G-agarose beads (Santa Cruz Biotechnology) were then added to the antigen-antibody complexes and incubated for 2 h at 4 °C. After extensive washing with IP buffer, the immunoprecipitated complexes were eluted and subjected to immunoblotting analysis. The following antibodies were used for immunoprecipitation: anti-TRIM21 (ab207728, Abcam), anti-SLC7A11 (ab307601, Abcam), anti-HA (ab18181, Abcam), with anti-IgG (ab172730, Abcam) serving as the negative control.

### 2.9 Protein stability assessment

To evaluate the protein stability of SLC7A11, transfected cells were pretreated with the proteasome inhibitor MG132 (20 μM, MedChemExpress) for 8 h, followed by exposure to cycloheximide (CHX, 100 μg/mL, MedChemExpress, USA) to inhibit *de novo* protein synthesis. SLC7A11 protein levels were subsequently analyzed by immunoblotting at designated time points (0, 6, 12, 18, and 24 h) after CHX treatment.

### 2.10 Statistical analysis

All statistical analyses were performed using GraphPad Prism 7 (GraphPad Software, San Diego, CA, USA). Continuous variables are expressed as mean ± standard deviation (SD). Comparisons between two groups were conducted using Student’s *t*-test, while comparisons among multiple groups were analyzed by one-way analysis of variance (ANOVA) followed by Tukey’s post hoc test. A p-value of less than 0.05 was considered statistically significant.

## 3 Results

### 3.1 TRIM21 is significantly upregulated in both clinical IVDD samples and oxidative stress-induced NPCs

**Figure 1:**
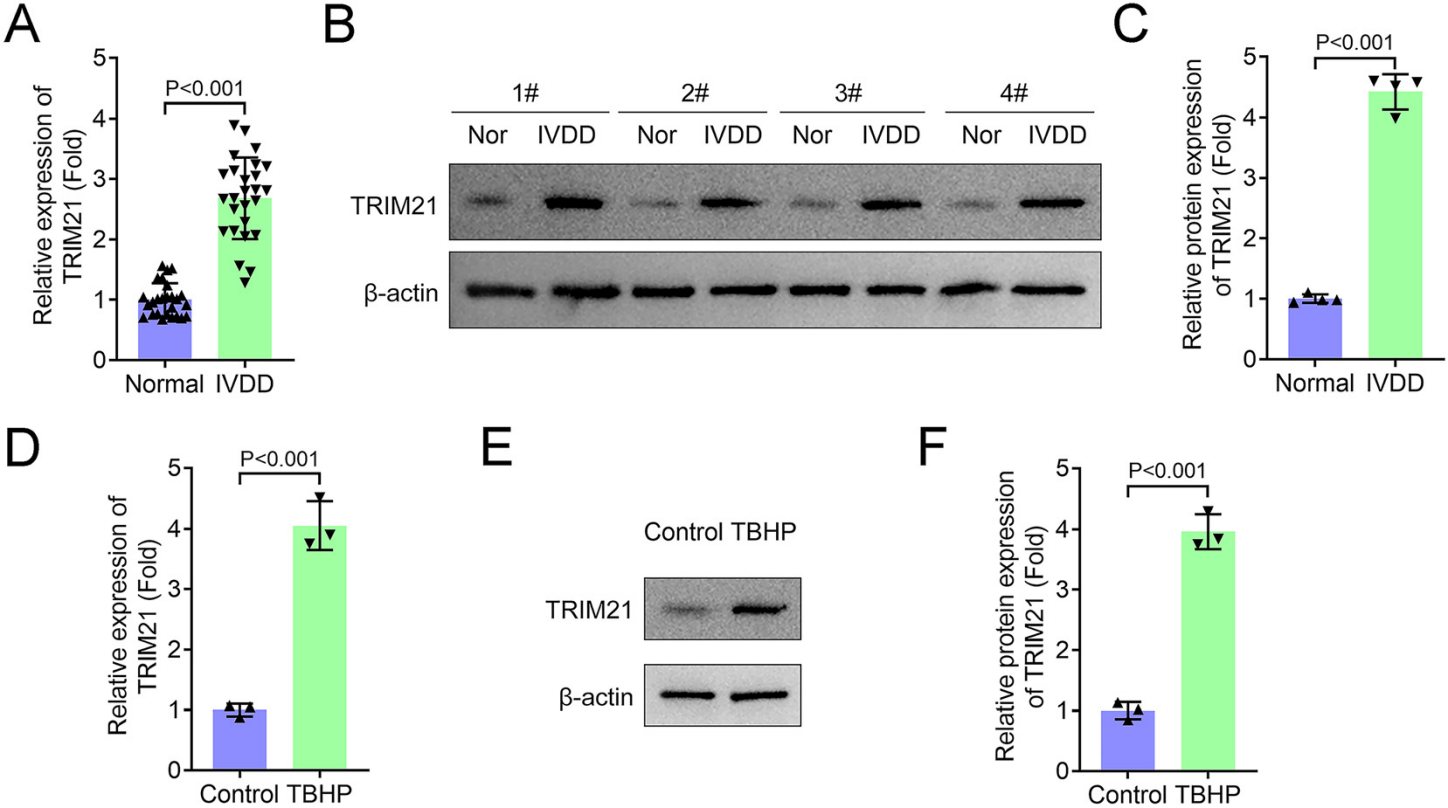
TRIM21 is significantly upregulated in both clinical IVDD samples and oxidative stress-induced NPCs. (A) The mRNA level of TRIM21 in serum of patients with IVDD was detected by qPCR. (*n* = 26 independent biological replicates/group). (B–C) The protein expression of TRIM21 in serum of patients with IVDD was detected by western blot. C: Quantification of TRIM21 protein levels. (*n* = 4 independent biological replicates/group). (D) The mRNA level of TRIM21 in TBHP-treated NPCs was detected by qPCR. (E–F) The protein expression of TRIM21 in TBHP-treated NPCs was detected by western blot. F: Quantification of TRIM21 protein levels. All data are expressed as the means ± SD. (*n* = 3 independent biological replicates/group *in vitro* experiments). Data were analyzed by Student’s *t*-test.

In this study, we first examined the expression pattern of TRIM21 in patients with IVDD compared to healthy controls. Both qPCR and western blot analyses revealed a significant upregulation of TRIM21 in the serum of IVDD patients (Figure 1A–C). To further investigate this observation, we established an *in vitro* model by treating NPCs with TBHP. Consistent with the clinical data, TRIM21 expression was markedly increased in TBHP-stimulated NPCs at both mRNA and protein levels (Figure 1D–F). These results collectively indicate that TRIM21 is involved in the pathogenesis of disc degeneration.

### 3.2 TRIM21 knockdown inhibits TBHP-induced ferroptosis in NPCs

**Figure 2:**
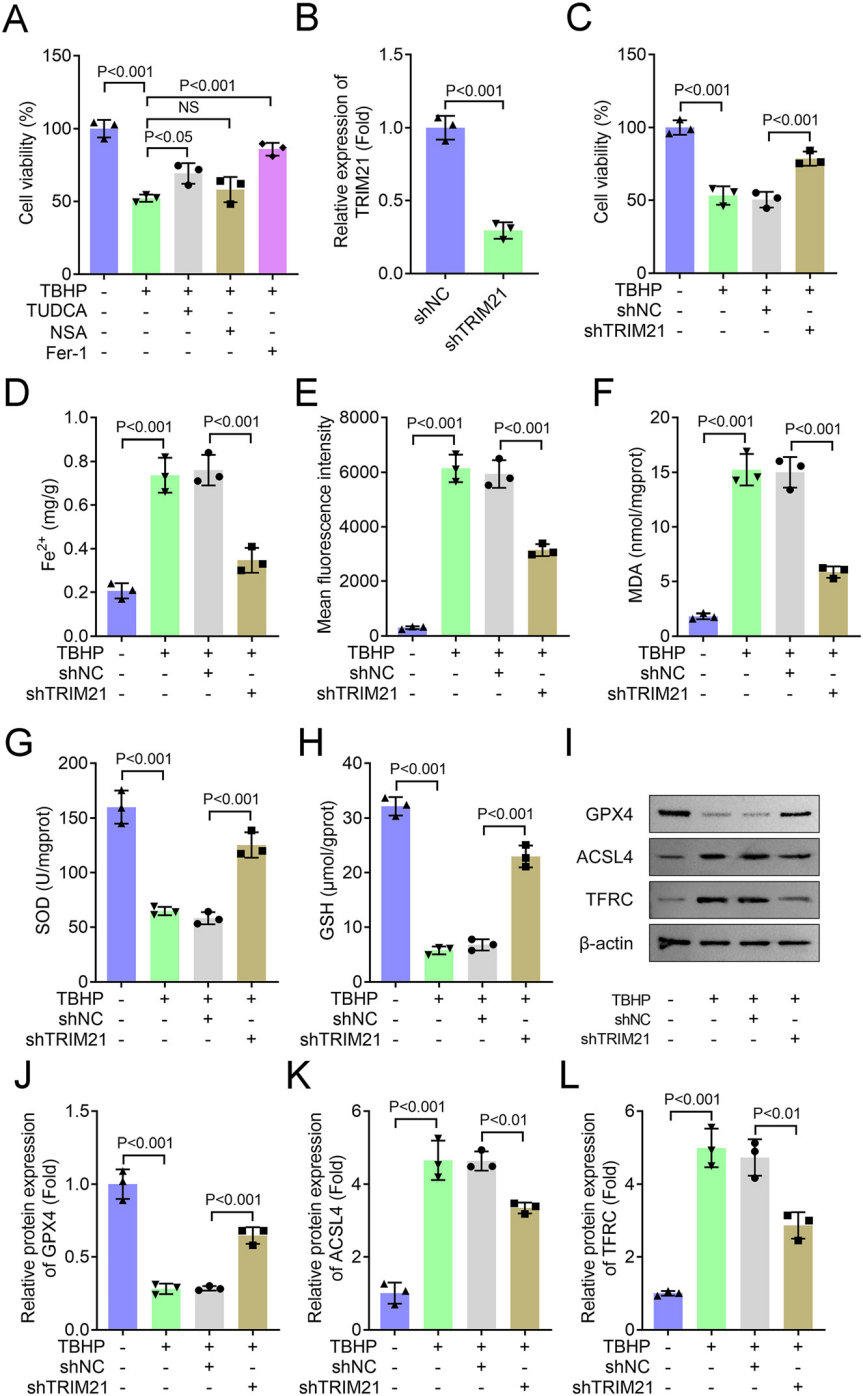
TRIM21 knockdown inhibits TBHP-induced ferroptosis in NPCs. (A) Cell viability was measured using the CCK-8 assay. (B) The mRNA level of TRIM21 was detected by qPCR. Student’s *t*-test. (C) Cell viability was measured using the CCK-8 assay. (D–H) Levels of Fe^2+^, ROS, MDA, SOD, and GSH were detected using commercial assay kits. (I–L) The expression of ferroptosis-related proteins (GPX4, ACSL4, and TFRC) was analyzed by Western blot. J–L: Quantification of GPX4, ACSL4, and TFRC protein levels. All data are expressed as the means ± SD. (*n* = 3 independent biological replicates/group *in vitro* experiments). Data were analyzed by one-way ANOVA with Tukey’s test for post hoc comparisons.

To investigate the biological function of TRIM21, we first identified the predominant form of cell death induced by TBHP in NPCs. Cells were treated with TBHP in the presence of specific inhibitors of apoptosis (TUDCA), necroptosis (NSA), or ferroptosis (Fer-1). As shown in Figure 2A, TBHP stimulation significantly decreased NPC viability. This effect was substantially reversed by both TUDCA and Fer-1, whereas NSA showed no significant protective effect. Since Fer-1 demonstrated the most pronounced rescue of cell viability, we subsequently focused on the ferroptosis pathway.

We next knocked down TRIM21 expression in NPCs using shRNA, which was confirmed at the protein level (Figure 2B). TRIM21 knockdown effectively attenuated the TBHP-induced reduction in cell viability (Figure 2C). Consistent with a ferroptosis phenotype, TBHP treatment significantly elevated intracellular levels of Fe^2+^, ROS, and MDA – all of which were markedly suppressed by TRIM21 silencing (Figure 2D–F). Furthermore, the TBHP-induced depletion of the antioxidants SOD and GSH was restored upon TRIM21 knockdown (Figure 2G and H). Western blot analysis of ferroptosis-related proteins showed that TBHP-mediated downregulation of GPX4 and upregulation of ACSL4 and TFRC were significantly reversed by TRIM21 knockdown (Figure 2I–L). Collectively, these results demonstrate that TRIM21 knockdown alleviates TBHP-induced ferroptosis in NPCs by restoring redox homeostasis and modulating key ferroptosis-related proteins, underscoring TRIM21 as a critical regulator of oxidative stress-induced cell death in disc degeneration.

### 3.3 Ferrostatin-1 rescues TRIM21-overexpression-induced ferroptotic damage in TBHP-stimulated NPCs

**Figure 3:**
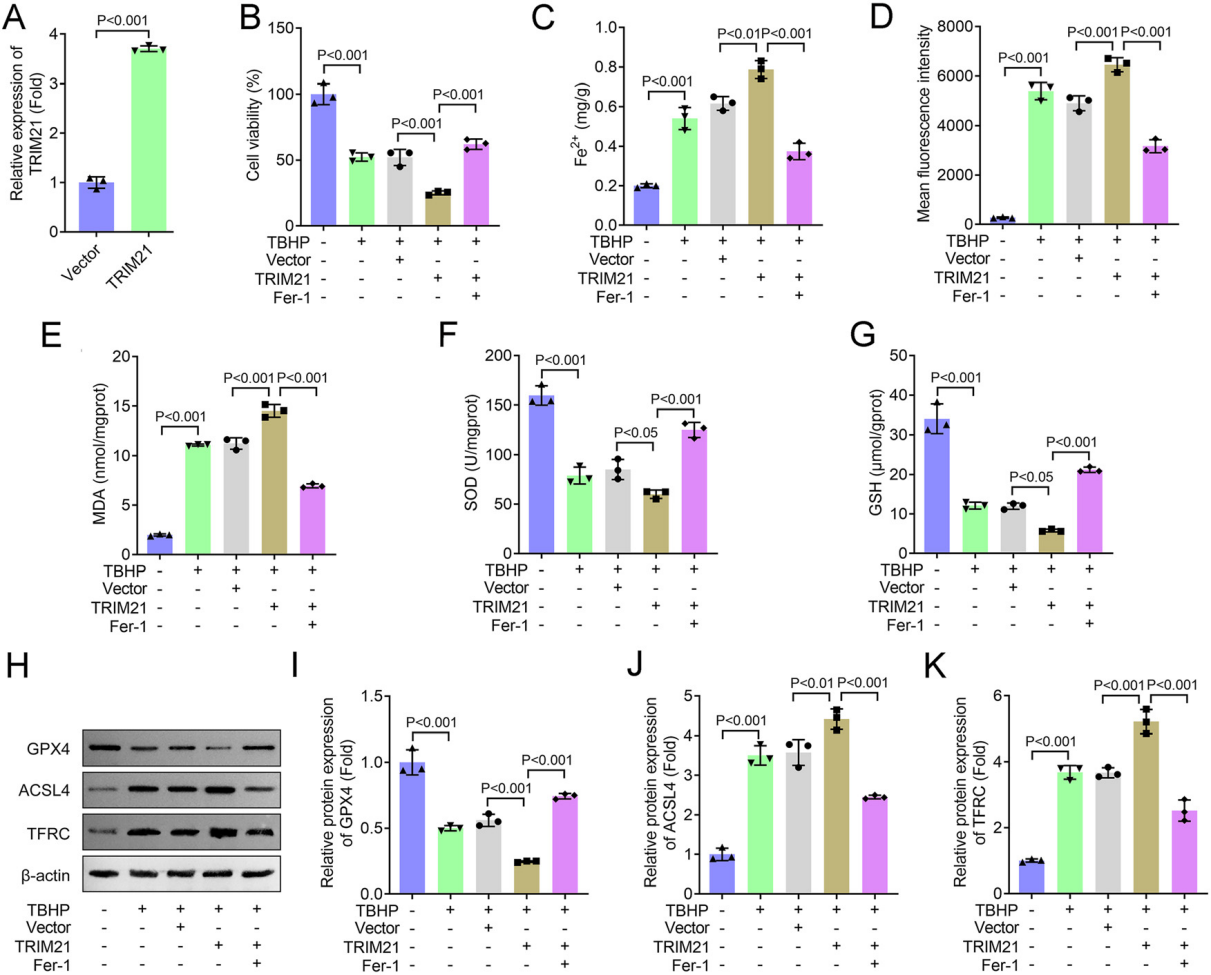
Ferrostatin-1 rescues TRIM21-overexpression-induced ferroptotic damage in TBHP-stimulated NPCs. (A) The mRNA level of TRIM21 was detected by qPCR. Student’s *t*-test. (B) Cell viability was measured using the CCK-8 assay. (C–G) Levels of Fe^2+^, ROS, MDA, SOD, and GSH were detected using commercial assay kits. (H–K) The expression of ferroptosis-related proteins (GPX4, ACSL4, and TFRC) was analyzed by Western blot. I–K: Quantification of GPX4, ACSL4, and TFRC protein levels. All data are expressed as the means ± SD. (*n* = 3 independent biological replicates/group *in vitro* experiments). Data were analyzed by one-way ANOVA with Tukey’s test for post hoc comparisons.

To determine whether the effects of TRIM21 overexpression in NPCs are specifically mediated through ferroptosis, we treated TBHP-stimulated NPCs with the ferroptosis inhibitor Fer-1. As shown in Figure 3A, successful TRIM21 overexpression was confirmed. Under TBHP stimulation, TRIM21 overexpression significantly reduced cell viability, an effect that was effectively rescued by Fer-1 treatment (Figure 3B). Furthermore, Fer-1 markedly attenuated the TRIM21-induced accumulation of Fe^2+^ (Figure 3C) and suppressed ROS overproduction (Figure 3D). Lipid peroxidation, as assessed by MDA levels, was significantly elevated by TRIM21 overexpression under TBHP treatment, and this increase was reversed by Fer-1 (Figure 3E). Additionally, Fer-1 restored the activities of the antioxidant defenses SOD (Figure 3F) and GSH (Figure 3G), both of which were impaired by TRIM21 overexpression. At the molecular level, TRIM21 downregulated the anti-ferroptotic protein GPX4 and upregulated the pro-ferroptotic proteins ACSL4 and TFRC under TBHP stimulation; these alterations were consistently reversed by Fer-1 treatment (Figure 3H–K). In summary, these findings demonstrate that TRIM21 promotes IVDD pathogenesis through a ferroptosis-dependent mechanism, specifically by modulating key ferroptotic regulators and lipid peroxidation signaling.

### 3.4 TRIM21 decreases the expression of SLC7A11 by promoting the K48-linked ubiquitination of SLC7A11

**Figure 4:**
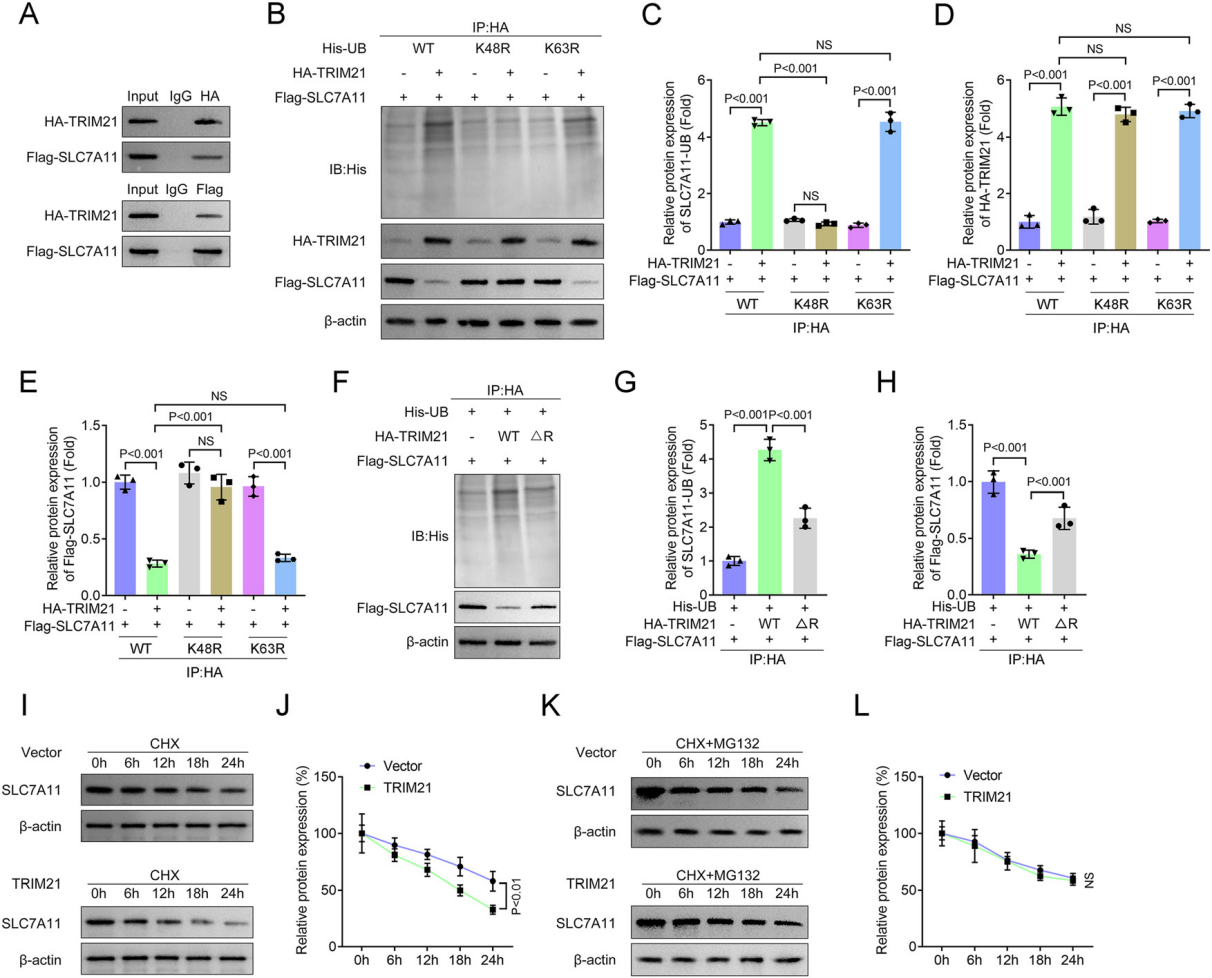
TRIM21 decreases the expression of SLC7A11 by promoting the K48-linked ubiquitination of SLC7A11. (A) The interaction between TRIM21 and SLC7A11 proteins was analyzed by Co-IP. (B–E) NPCs were transfected with Flag-SLC7A11, HA-TRIM21, and WT/K48R/K63R His-UB. After IP with HA, immunoblotting was performed with anti-His. C–E: Quantification of SLC7A11-UB, HA-TRIM21, and Flag-SLC7A11 protein levels. (F–H) NPCs were transfected with His-UB, Flag-SLC7A11, and WT TRIM21 or MUT TRIM21. After IP with HA, immunoblotting was performed with anti-His. G–H: Quantification of SLC7A11-UB and Flag-SLC7A11 protein levels. (I–J) NPCs were transfected with TRIM21 overexpression plasmids or empty vectors, and treated with CHX for 0, 6, 12, 18, and 24 h. Protein levels of SLC7A11 were detected using immunoblotting. (K–L) NPCs were transfected with TRIM21 overexpression plasmids or empty vectors, treated with MG132, and then stimulated with CHX for 0, 6, 12, 18, and 24 h. Protein levels of SLC7A11 were detected using immunoblotting. All data are expressed as the means ± SD. (*n* = 3 independent biological replicates/group *in vitro* experiments). Data were analyzed by one-way or two-way ANOVA with Tukey’s test for post hoc comparisons.

SLC7A11 is well-established as a central regulator of ferroptosis, primarily through its role in cystine uptake and GSH biosynthesis. To elucidate the molecular mechanism by which TRIM21 regulates ferroptosis, we first performed Co-IP assays, which confirmed a direct physical interaction between TRIM21 and SLC7A11 (Figure 4A). Subsequent ubiquitination analysis revealed that TRIM21 overexpression significantly enhanced K48-linked polyubiquitination of SLC7A11, while K63-linked ubiquitination remained largely unaffected (Figure 4B–E). Furthermore, HA-TRIM21-wt markedly promoted SLC7A11 ubiquitination and reduced its protein expression, whereas a TRIM21-mut lacked this effect (Figure 4F–H). In CHX chase assays, TRIM21 overexpression accelerated the degradation of SLC7A11 (Figure 4I and J), which was effectively blocked by the proteasome inhibitor MG132 (Figure 4K and L). These findings collectively demonstrate that TRIM21 promotes K48-linked polyubiquitination and subsequent proteasomal degradation of SLC7A11, uncovering a novel ubiquitination-dependent mechanism through which TRIM21 regulates ferroptosis in IVDD.

### 3.5 Knockdown of SLC7A11 reverses the inhibitory effects of TRIM21 knockdown on TBHP-induced ferroptosis in NPCs

**Figure 5:**
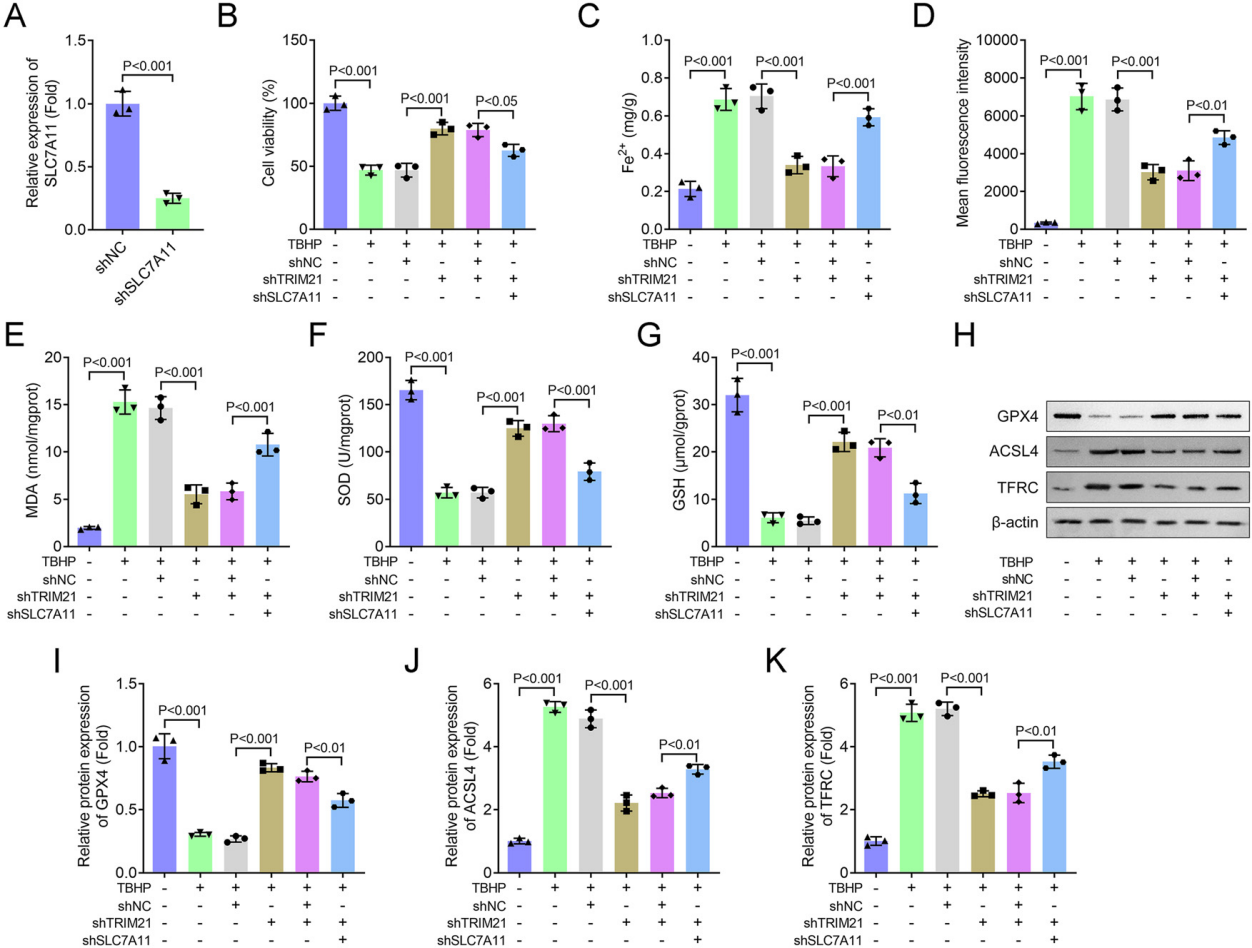
Knockdown of SLC7A11 reverses the inhibitory effects of TRIM21 knockdown on TBHP-induced ferroptosis in NPCs. (A) The mRNA level of SLC7A11 was detected by qPCR. Student’s *t*-test. (B) Cell viability was measured using the CCK-8 assay. (C–G) Levels of Fe^2+^, ROS, MDA, SOD, and GSH were detected using commercial assay kits. (H–K) The expression of ferroptosis-related proteins (GPX4, ACSL4, and TFRC) was analyzed by Western blot. I–K: Quantification of GPX4, ACSL4, and TFRC protein levels. All data are expressed as the means ± SD. (*n* = 3 independent biological replicates/group *in vitro* experiments). Data were analyzed by one-way ANOVA with Tukey’s test for post hoc comparisons.

To determine whether the biological functions of TRIM21 are mediated through SLC7A11, we performed a series of rescue experiments. First, SLC7A11 expression was effectively knocked down in NPCs via shRNA transfection (Figure 5A). We found that while TRIM21 knockdown significantly improved the viability of TBHP-stimulated NPCs, this protective effect was largely abolished by concurrent SLC7A11 silencing (Figure 5B). Moreover, the suppressive effects of TRIM21 knockdown on TBHP-induced accumulation of Fe^2+^, ROS, and MDA were also reversed upon SLC7A11 downregulation (Figure 5C–E). Similarly, the enhanced activities of the antioxidant enzymes SOD and GSH resulting from TRIM21 knockdown were compromised by SLC7A11 silencing (Figure 5F and G). At the protein level, the upregulation of GPX4 and downregulation of ACSL4 and TFRC induced by TRIM21 knockdown were negated in the absence of SLC7A11 (Figure 5H–K). Taken together, these results demonstrate that the anti-ferroptotic effects of TRIM21 knockdown are SLC7A11-dependent, establishing SLC7A11 as an essential downstream effector of TRIM21 in the regulation of oxidative stress and ferroptosis in NPCs.

## 4 Discussion

IVDD is a leading cause of chronic low back pain, in which oxidative stress-induced NPC death plays a central pathogenic role [18]. This study provides compelling evidence that TRIM21 significantly contributes to IVDD by promoting ferroptosis in NPCs via ubiquitin-mediated degradation of SLC7A11. Our findings reveal a novel molecular pathway connecting oxidative stress, protein ubiquitination, and iron-dependent cell death in the progression of IVDD.

First, we demonstrated that TRIM21 expression is significantly upregulated in clinical IVDD specimens and in TBHP-treated NPCs, suggesting that TRIM21 may act as a molecular sensor and effector in the pathological microenvironment of disc degeneration. This observation aligns with growing evidence that E3 ubiquitin ligases – including several TRIM family members – are dysregulated in degenerative diseases [19], including IVDD [20]. For instance, TRIM8 knockdown in chondrocytes has been shown to suppress IL-1β-induced inflammatory responses in osteoarthritis [21], while Liu et al. reported that TRIM21 deficiency alleviates osteoporosis in murine models [22]. Notably, Zheng et al. revealed that TRIM21 is upregulated in nucleus pulposus tissues in correlation with IVDD severity, and its deletion protected NP cells from oxidative stress-induced degeneration and attenuated age-related IVDD in mice [23]. In our study, the increased TRIM21 expression under TBHP-induced oxidative stress further supports its role as a stress-responsive regulator in NPCs. Given that excessive ROS accumulation is a hallmark of IVDD and a well-established driver of disease progression, our findings reinforce the view that TRIM21 upregulation may exacerbate disc degeneration by modulating oxidative damage responses.

The identification of ferroptosis as the predominant form of cell death in TBHP-treated NPCs represents a key advance in understanding IVDD pathophysiology. Our comparative analysis of cell death inhibitors showed that the ferroptosis inhibitor Fer-1 exerted a more pronounced protective effect on NPCs under oxidative stress than inhibitors of apoptosis (TUDCA) or necroptosis (NSA). This observation aligns with recent studies highlighting the involvement of ferroptosis in IVDD [24], 25], justifying our focus on targeting ferroptosis-specific pathways for therapeutic intervention. Most notably, TRIM21 knockdown effectively counteracted all major hallmarks of ferroptosis [26], including: restored cell viability, reduced intracellular Fe^2+^ accumulation, suppressed ROS and MDA production, and recovered antioxidant capacity (reflected by GSH and SOD levels). Moreover, TRIM21 silencing normalized the expression of key ferroptosis-related proteins, reversing TBHP-induced downregulation of GPX4 and upregulation of ACSL4 and TFRC. These comprehensive findings establish TRIM21 as a central regulator of ferroptosis in NPCs under oxidative stress, extending its functional repertoire beyond previously recognized roles in immune regulation and antiviral defense [27]. Furthermore, the observation that Ferrostatin-1 effectively rescued TRIM21-overexpression-induced ferroptotic damage underscores the specificity of TRIM21 in driving ferroptosis rather than other forms of cell death. This was evidenced by the restoration of cell viability, attenuation of Fe^2+^ and ROS accumulation, reversal of lipid peroxidation, and normalization of key ferroptotic markers upon Fer-1 treatment. These data strongly support the conclusion that TRIM21 exacerbates IVDD primarily through a ferroptosis-dependent pathway, thereby highlighting the potential of targeting this axis for therapeutic intervention.

E3 ubiquitin ligases orchestrate diverse biological processes by catalyzing distinct ubiquitin chain linkages, such as K48, K63, and K11 [28]. Canonical K48-linked polyubiquitination typically targets substrates for proteasomal degradation, thereby regulating cell cycle progression, inflammatory responses, and misfolded protein clearance. In contrast, K63-linked polyubiquitination primarily mediates non-degradative signaling, modulating processes such as DNA damage repair, endocytosis, and NF-κB pathway activation by altering protein interactions or subcellular localization [29], 30]. A central mechanistic finding of this study is that TRIM21 physically interacts with SLC7A11 – a core component of the cystine/glutamate antiporter system Xc− – and promotes its proteasomal degradation via K48-linked polyubiquitination. This finding is particularly significant given that SLC7A11 serves as a master suppressor of ferroptosis, maintaining cellular redox homeostasis by facilitating cystine uptake for glutathione synthesis [31]. Previous studies have shown that SLC7A11 is subject to ubiquitination, and several proteins – including SOCS2, CRL3, and BAP1 – have been reported to regulate ferroptosis through modulating SLC7A11 ubiquitination [32], [33], [34]. Notably, TRIM3 has been shown to promote SLC7A11 ubiquitination and degradation, thereby accelerating ferroptosis in lung cancer [35]. However, the regulatory relationship between TRIM21 and SLC7A11 has remained unexplored. In this study, we observed that wild-type TRIM21, but not its E3 ligase-deficient mutant, promoted SLC7A11 degradation, highlighting the essential role of TRIM21’s catalytic activity in this process. These results reveal a previously unrecognized post-translational regulatory axis in which TRIM21-mediated SLC7A11 degradation sensitizes NPCs to ferroptosis under oxidative stress. Furthermore, rescue experiments unequivocally established SLC7A11 as the critical downstream effector of TRIM21 in ferroptosis regulation. Although TRIM21 knockdown conferred strong protection against TBHP-induced ferroptosis, concurrent SLC7A11 silencing abolished this effect, reinstating iron overload, lipid peroxidation, and antioxidant depletion. These results are consistent with prior reports that SLC7A11 deficiency exacerbates ferroptosis by impairing glutathione-dependent antioxidant defenses [36], 37]. Importantly, our study identifies TRIM21 as an upstream negative regulator of SLC7A11 in IVDD, providing a mechanistic explanation for how oxidative stress triggers ferroptosis in NPCs.

This study has several limitations. First, the research was primarily conducted using *in vitro* cell models, which may not fully recapitulate the complex tissue microenvironment of human intervertebral discs. Second, we focused specifically on the role of ferroptosis without exploring potential crosstalk with other forms of cell death that may also contribute to IVDD progression. Third, the absence of *in vivo* validation using animal models limits the translational relevance of our findings. Finally, the upstream regulatory mechanisms controlling TRIM21 expression in IVDD remain to be elucidated.

In conclusion, our study delineates a previously unrecognized pathway through which TRIM21 promotes IVDD progression by facilitating SLC7A11 degradation and subsequent ferroptosis in NPCs. The TRIM21-SLC7A11 regulatory axis disrupts cellular redox homeostasis, leading to iron overload, lipid peroxidation, and ultimately NPC death. These findings significantly advance our understanding of the molecular mechanisms underlying disc degeneration and provide a conceptual framework for developing novel therapeutic strategies targeting the TRIM21-SLC7A11-ferroptosis axis.
